# Host and Bacterial Iron Homeostasis, an Underexplored Area in Tuberculosis Biomarker Research

**DOI:** 10.3389/fimmu.2021.742059

**Published:** 2021-10-29

**Authors:** Lucinda Baatjies, Andre G. Loxton, Monique J. Williams

**Affiliations:** ^1^ Department of Science and Innovation (DSI)-National Research Foundation (NRF) Centre of Excellence for Biomedical Tuberculosis Research, South African Medical Research Council Centre for Tuberculosis Research, Division of Molecular Biology and Human Genetics, Faculty of Medicine and Health Sciences, Stellenbosch University, Cape Town, South Africa; ^2^ Department of Molecular and Cell Biology, University of Cape Town, Cape Town, South Africa

**Keywords:** tuberculosis, diagnosis, biomarkers, Fe-S clusters, siderophores, iron

## Abstract

*Mycobacterium tuberculosis* (Mtb) “a human adapted pathogen” has found multiple ways to manipulate the host immune response during infection. The human immune response to Mtb infection is a highly complex cascade of reactions, with macrophages as preferred intracellular location. Interaction with the host through infection gives rise to expression of specific gene products for survival and multiplication within the host. The signals that the pathogens encounter during infection cause them to selectively express genes in response to signals. One strategy to identify Mtb antigens with diagnostic potential is to identify genes that are specifically induced during infection or in specific disease stages. The shortcomings of current immunodiagnostics include the failure to detect progression from latent infection to active tuberculosis disease, and the inability to monitor treatment efficacy. This highlights the need for new tuberculosis biomarkers. These biomarkers should be highly sensitive and specific diagnosing TB infection, specifically distinguishing between latent infection and active disease. The regulation of iron levels by the host plays a crucial role in the susceptibility and outcome of Mtb infection. Of interest are the siderophore biosynthetic genes, encoded by the *mbt-1* and *mbt*-2 loci and the SUF (mobilization of sulphur) operon (*sufR-sufB-sufD-sufC-csd-nifU-sufT*), which encodes the primary iron-sulphur cluster biogenesis system. These genes are induced during iron limitation and intracellular growth of Mtb, pointing to their importance during infection.

## Introduction

TB disease is a major cause of poor health even in the 20^th^ century. In 2019, approximately 10 million people fell ill with TB, while 1.2 million deaths amongst HIV-negative individuals and 208 000 deaths amongst HIV-positive individuals are estimated (1).

The innocuous or asymptomatic nature of TB infection contributes to its elusiveness (2). In individuals where the host immune response contains the infection, Mtb has the ability to establish a latent TB infection (LTBI), allowing long persistence of viable bacilli in the host without any clinical symptoms (3, 4). Following exposure to Mtb, 90-95% of infected individuals remain healthy (asymptomatic) and are latently infected (5). Between 3-10% of all LTBI individuals develop active disease during their lifespan (6). LTBI individuals therefore represents a long-lived pool from which active TB disease will continue to develop (7, 8). The risk of developing active disease decreases exponentially over time, the first 2 years after infection being crucial with an 80% chance of developing TB (2).

Where TB does develop, the non-specific nature of disease often delays diagnosis and treatment, increasing the risk of infecting others (9). A further challenge facing global TB control is the emergence and spread of multi-drug resistant Mtb (MDR-TB) strains. MDR-TB is defined as infection with an Mtb strain that is resistant to, at least, rifampicin and isoniazid (10). In 2017, an estimated 558 000 individuals developed drug-resistant TB and 230 000 died (11). Alarmingly, more than a third of the MDR cases are not enrolled for MDR treatment, with this percentage approaching two-thirds in some areas in Asia and Africa (2).

## Iron Homeostasis and Mtb Pathogenesis

Iron is an essential micronutrient which is important for the host and Mtb metabolic processes (12, 13). The regulation of iron levels by the host influences the susceptibility to and outcome of Mtb infection (14, 15), since high macrophage iron stores and nutritional iron overload are associated with a higher chance of developing TB and increased severity of the disease (16–18). Most of the iron in the human body is stored as protein-bound heme (70-75%), while the remainder is associated with the plasma transport proteins transferrin and lactoferrin or bound to the intracellular storage protein, ferritin (19). Since intestinal absorption provides less than 10% of the host’s iron requirement, macrophages play a key role in maintaining serum levels by recycling iron from aged erythrocytes (20). Ferroportin (FPN-1) exports iron from macrophages into the bloodstream and is therefore central to maintaining iron homeostasis. During infection, inflammatory cytokines, primarily IL-6, induce expression of the hormone hepcidin to trigger FPN-1 internalization, preventing iron export (21); a response which can lead to anemia in chronic infections like TB (22). While macrophages represent a significant iron reservoir in the host (23, 24), inflammatory cytokines also induce ferritin synthesis in macrophages thereby reducing the labile iron pool.

**Figure 1:**
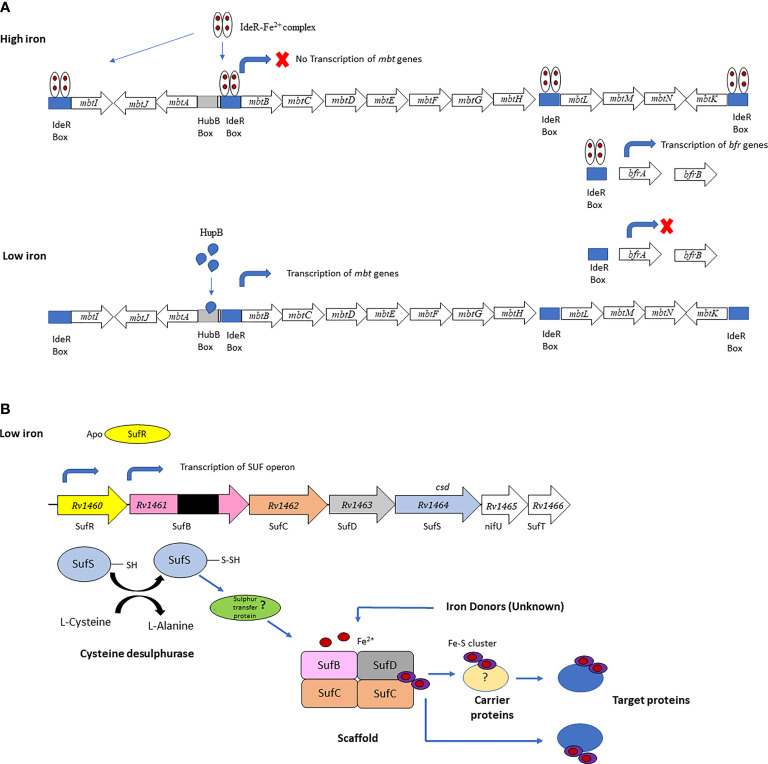
**(A)** IderR regulates Mtb siderophore biosynthesis and iron storage. The *mbtA-J* genes synthesize the siderphore core and the *mbtL-K* genes synthesize the mbtK-acyl complex. Transcription of the siderphore biosynthesis genes is inhibited under iron-sufficient conditions and synthesis of the iron storage proteins is activated when the IdeR-Fe^2+^ complex binds to the IdeR boxes in the mbt locus and upstream of the *bfrA* gene. Under iron limiting conditions the IdeR-Fe^2+^ complex does not form and does not bind to the IdeR box in the *mbtB* locus, allowing HupB to bind to the hupB box upstream of the IdeR box activating transcription of the *mbt* genes. In the absence of the IdeR-Fe^2+^ complex transcription of the iron storage genes *bfrA* and *bfrB* is repressed. Panel **(A)** is adapted from Sritharan (12). **(B)** Fe-S biogenesis machinery in Mtb encoded by a single operon (*sufR-sufB-sufD-sufC-csd-nifU-sufT*). All the genes in the operon, except for *sufR*, were predicted to be essential by forward genetic screens. This figure is adapted from Willemse (29).

Mtb employs several mechanisms to acquire iron from the host during infection, including siderophores (mycobactin and carboxymycobactin), which sequester iron from host iron-binding proteins, heme-binding proteins and specialized iron transporters (23, 25). The genes encoding the core Mtb siderophore biosynthetic machinery are organized in two clusters, *mbt-1*, and the *mbt-2* loci. The *mbt-1* locust consists of 10 genes *mbtA - mbtJ* and *mbt-2* locus consist of 4 genes *mbtN – mbtK* (12, 26). An *mbtB* Mtb mutant, defective in the first step of mycobactin synthesis, is impaired for growth in low-iron media and macrophages (25, 27), highlighting the essential role of siderophores in obtaining iron during intracellular growth (26). This is consistent with the induction of *mtbA-J* during growth in IFN-γ activated macrophages and increased *mbtB* mRNA levels detected during Mtb infection in mice (16). The *mbt* operon is downregulated under iron replete conditions by the iron-dependent regulator (IdeR) (28), which concomitantly induces the iron storage genes *bfrA (*bacterioferritin) and *bfrB* (ferritin like protein) (26). The transcription of the *mbt* and *bfr* genes are also dependent on HupB, a 28-kDa DNA-binding protein which is induced under iron-limiting conditions. Binding of HupB to the *mbtB* is iron-dependent and is thought to induce expression in the absence of IdeR binding (**Figure 1A**) (28). HupB was identified in the cell wall fraction of Mtb, and recently a novel role in the transfer of iron between extracellular ferri-carboxymycobactin and membrane-bound mycobactin was proposed (30). A *hupB* knockout strain of Mtb showed a marked reduction in mycobactin and carboxymycobactin levels and was unable to survive in macrophages (28), likely due to impaired iron acquisition during intracellular growth.

A recent study by Pisu et al. (31) demonstrated a correlation between bacterial replication and iron availability in the mouse model of infection. Using dual RNA-seq they showed two distinct host and bacterial transcriptional signatures for alveolar, and monocyte-derived interstitial macrophages. Alveolar macrophage-derived bacteria had high expression of genes suggestive of iron replete conditions, e.g. iron storage proteins (*BfrB*), while bacteria derived from interstitial macrophages had high expression of genes associated with iron limitation, such as siderophore biosynthesis (31). In agreement with the bacterial response, interstitial macrophages expressed genes associated with iron sequestration, while alveolar macrophages showed a phenotype associated with iron release. Notably, alveolar macrophages were more permissive for bacterial growth than interstitial macrophages (31). A study investigating Mtb disease progression in knockout mice lacking the ferritin heavy chain (FtH) in myeloid-derived cell populations (32) demonstrated the importance of host iron homeostasis in disease. *Fth^-/-^* mice showed increased bacterial organ burden, increased bacterial dissemination, excessive inflammation, and decreased survival due to altered immune cell metabolism. Furthermore, Mtb infection was shown to impair iron absorption and alter host iron homeostasis. The observation that iron levels and deposition patterns are altered in lung tissue from human TB patients suggested that these finding have relevance for human disease (32).

## The Current Tuberculosis Diagnostic Paradigm

The existing diagnostic tools for TB have several limitations. The most important criteria for confirming TB diagnosis are acid-fast bacilli (AFB) in sputum smears, radiographic signs, clinical symptoms and risk factors, or a combination of these (33). The gold standard is the detection of Mtb in human specimens by microbiological culture, which has a high sensitivity for detecting Mtb, but requires 2 to 6 weeks for interpretation. Sputum smear microscopy is an inexpensive tool, which is rapid and simple for diagnosing pulmonary TB, however it has low and variable sensitivity when the mycobacterial load is low, when the site of infection is not easily accessible, or when patients fail to produce a sputum sample (34). These limitations result in reduced success in identifying individuals at risk for developing extrapulmonary TB (EPTB), pediatric TB and pulmonary TB in people living with HIV (35).

Chest X-rays are also used in diagnosis, but can present with atypical radiological finding that are indistinguishable from community–acquired pneumonia (36). The intradermal purified protein derivative (PPD) test or tuberculin test (TST), which detects an immune response to bacterial proteins, is limited by the fact that it is not Mtb-specific, since it shares Mtb antigens with other mycobacterial strains (37). Another logistical limitation of the TST test is that a second patient visit is required to evaluate the test results (38). The Interferon Gamma Release Assay (IGRA) measures the levels of interferon-gamma (IFN-γ) released from T-lymphocytes after *in vitro* stimulation with the Mtb-specific antigens, early secreted antigenic target 6 kDa (ESAT-6; Rv3875), and culture filtrate protein 10 kDa (CFP-10; Rv3874) (38, 39). It has the advantage over TST in that it uses antigens in the region of difference-1 (RD-1), found only in Mtb, thereby erasing cross-reactivity with the BCG vaccine strain. IGRA as diagnostic tool however fails to differentiate between LTBI and active TB and has limited predictive value, failing to identify individuals at risk of progressing to active TB (40–42).

The most recent and widely implemented new diagnostic test, namely the GeneXpert MTB/RIF, has revolutionized the diagnosis of TB. Xpert MTB/RIF is an automated molecular test, using real-time polymerase chain reaction (PCR) to amplify the *rpoB* gene (Mtb specific sequence), and probe it with molecular beacons for mutations within the rifampicin resistance determining region (RRDR) (43). It detects 75% of smear negative pulmonary TB cases, as well as rifampicin resistance with results within 2 hours. Although GeneXpert MTB/RIF improves TB case detection, two recent randomized trials in Southern Africa suggest that on its own, GeneXpert may not be able to significantly reduce tuberculosis-related morbidity and mortality (44, 45).

## Advances in Mtb Biomarker Research and Why They Are Not Good Enough

Improved TB control requires the development of sensitive and specific diagnostic tests for the identification of TB infection, and specifically to distinguishing between latent and active disease (46). Differential expression of immunological markers by the host in response to Mtb infection may enable us to distinguish the stages of TB infection and disease, and therefore hold promise as diagnostic biomarkers (47). Multiplex assays and advances in ‘omics’ technology have facilitated the study of immune biomarkers in samples that are easy to access, such as peripheral blood, saliva, or urine (48). In the following sections, we highlight studies aimed at identifying host biomarkers that differentiate between stages of Mtb infection and monitor disease progression. The use of infection-stage specific Mtb antigens in these assays is also discussed.

## Host Biomarkers That Differentiate Between the Different Stages of Mtb Infection and Monitor Disease Progression

Stimulating whole blood from Mtb-infected individuals with Mtb specific antigens causes the release of several host cytokines and chemokines (49). Several studies have sought to identify cytokines (**Table 1**) and chemokines that might better discriminate between LTBI and active TB disease (58). Chegou et al. (58), showed an increase in multiple host markers other than IFN-γ after stimulating whole blood cells from TB patients and household contacts (HHCs) with Mtb antigens hypothesized to be differentially expressed during different phases of Mtb infection. The most promising diagnostic candidates were IL-12(p40), IP-10, IL-10 and TNF-α, which were able to discriminate between TB cases and HHC with 100% sensitivity and specificity following stimulation with Rv0081 (58). Rv0081 is a Mtb protein in the dormancy survival (DosR) regulon, which is induced during oxygen limitation and is required for anaerobic survival (59). Using a training sample set (n = 491) Chegou et al. (51) identified a seven-serum host protein biosignature (C reactive protein (CRP), transthyretin, IFN-g, complement factor H, apolipoprotein-A1, inducible protein 10 and serum amyloid A) that could diagnose TB disease. In a test set (n = 210), the signature had a sensitivity of 93.8% (95% CI 84.0% to 98.0%), specificity of 73.3% (95% CI 65.2% to 80.1%), and positive predictive value (PPV) and negative predictive value (NPV) of 60.6% (95% CI 50.3% to 70.1%) and 96.4% (95% CI 90.5% to 98.8%), respectively, irrespective of HIV infection status or ethnicity. The biosignature correctly classified 74% (17/23) of patients who were smear-negative but culture-positive and 67% (6/9) of patients who were both smear-negative and culture-negative (51). Wang et al. (52) identified a six-cytokine biosignature that could distinguish between active TB and LTBI with high levels of accuracy. In the screening group (n = 88) IFN-γ, IP-10, and IL-1Ra were identified following stimulation with ESAT-6/CFP-10, while IP-10, VEGF, and IL-12 (p70) were measured in unstimulated samples. Validation of the six-cytokine biosignature in a biomarker validation cohort (n = 216) showed a sensitivity of 88.2% and specificity of 92.1% and in a clinical validation cohort (n = 194) a sensitivity of 85.7%, a specificity of 91.3% with an accuracy of 88.7% (52).

**Table 1:** Potential biomarkers that can distinguish between different stages of *M.tb* infection.

Reference	Stimulation	Potential biomarkers	Distinguishing between	Assay
Chegou et al. (50)	Rv0081	IL-12(p40), IP-10, IL-10, and TNF-α	LTBI *vs* active TB	Luminex assay on 7-day diluted WBA supernatants
Chegou et al. (51)	Unstimulated	CRP, transthyretin, IFN-γ, CFH, ApoA-1, IP-10, and SAA	Diagnosing active pulmonary TB in adults	Luminex assay on serum samples
Wang et al. (52)	ESAT-6/CFP-10 Unstimulated	IFN-γ, IP-10, IL-1Ra IP-10, VEGF, IL-12 (p70)	LTBI *vs* active TB	Luminex assay on 24-hour WBA supernatants
Zak et al. (53)	No stimulation	ANKRD22, APOL1, BATF2, ETV7, FCGR1A, FCGR1B, GBP1, GBP2, GBP4, GBP5, SCARF1, SEPT4, SERPING1, STAT1, TAP1, TRAFD1	Predicting risk of tuberculosis disease progression	RNA-Seq transcriptome analysis technology adapted to qRT-PCR
Jacobs et al. (54)	No stimulation	CRP, ferritin, SAP, MCP-1, A2M, fibrinogen, TPA	Diagnosing TB disease	Luminex assay on saliva supernatants
Cao et al. (39)	No stimulation	Rv2002, Rv1408, Rv0389, Rv2421c, Rv0248c, Rv2026c, Rv2716, Rv2097c, Rv2031c, Rv2906c, Rv2928	LTBI *vs* active TB	Serum profiling on Mtb proteome microarray and ELISA
Day et al. (55)	Unstimulated ESAT-6, CFP-10, TB10.4, HCMV pp65, PPD	PD-1 on antigen specific CD4 T	Bacterial load and treatment response	Whole blood ICS phenotyping assay; BAL sample processing and Proliferation assay and *in vitro* PD1/PDL1 blockade
van Rensburg et al. (56)	No stimulation	*FASLG* and *IL5RA*	Monitor treatment response	qRT-PCR on B-cells
Chegou et al. (50)	118 Mtb phase dependant antigens	Rv0867c, Rv2389c, Rv2450c, Rv1009 and Rv1884c	Active TB *vs* HHC	IFN-g ELISA assay on 7-day diluted WBA
Loxton et al. (57)	HBHA	Multifunctional CD4+ T cells coexpressing INF-γ-, IL-2-, and IL-17-	Active TB *vs* HHC	IFN-g ELISA assay on 7-day diluted WBA

Transcriptional profiling of blood cells from TB, healthy uninfected and/or LTBI individuals has also shown promise in identifying TB biomarkers (60). A study by Zak et al. (53) identified a 16-gene blood transcriptional correlate of risk (COR) signature that predicted the risk of progression to TB in HIV-negative South African adolescents with LTBI with a sensitivity and specificity of 66.1% and 80.6% (with a 95% confidence interval (CI) 63.2 – 68.9 and 7.2 – 82.0), respectively, 12 months preceding TB. Validation in an independent group of adolescents from South African and Gambian cohorts reported a sensitivity of 53.7% (42.6 – 64.3) and a specificity of 82.8% (76.7–86) (53). Using saliva samples from suspected pulmonary TB individuals, Jacobs et al. (54) identified a seven-marker biosignature of CRP, ferritin, SAP, MCP-1, A2M, fibrinogen and TPA, that might be useful in the diagnosis of TB disease. TB disease was diagnosed with a sensitivity of 78.1% (95% CI, 59.6–90.1%) and specificity of 83.3% (95% CI, 72.3–90.7%) after leave-one-out cross validation (54). Cao et al. (39) screened for novel serum biomarkers that can discriminate between LTBI and active TB individuals using an Mtb proteome microarray containing 4,262 antigens. Significantly higher levels of 152 Mtb antigen-specific IgG antibodies was seen in the active TB compared to the LTBI group (p < 0.05). ELISA analysis of 11 candidate antigens were consistent with the microarray analysis. Antibodies specific for Rv2031c, Rv1408, and Rv2421c had highest areas under the curve (AUCs) of 0.8520, 0.8152, and 0.7970, respectively. The authors identified several antigens with potential as serum biomarkers for discriminating between active TB and LTBI (39).

Apart from diagnosis, biomarkers can be used for monitoring treatment response in TB patients. It is believed that people with active TB may have a flawed Mtb-specific T cell response due to the increasing bacterial load. One mechanism that gives rise to the inhibition of antigen-specific T cell effector function is the expression of inhibitory receptors such as PD-1, CTLA-4, LAG-3, TIM-3, and BTLA on CD4 T cells (55). Day et al. (55) showed the expression of programmed cell death protein 1 (PD-1) on Mtb specific CD4 T cells, but not CD8 T cells. The expression of PD-1 characterizes a population of effector cells, that have engaged their cognate antigen and have the capacity to produce Th1 cytokines. This study has provided novel insight into the function of PD-1 pathway in regulating T cell response during Mtb infection and the expression of PD-1 on antigen specific CD4 T cells as a biomarker for bacterial load and treatment response in human TB (55). A pilot study by van Rensburg et al. (56) evaluating the transcriptome of B-cells showed the downregulation of *FASLG* and *IL5RA* in TB cases at diagnosis compared to healthy controls (HCs) and the upregulation of these genes in TB cases after month 6 of treatment. These genes have the potential to be used as a biosignature to monitor treatment response but need to be validated in a larger cohort (56). Using an integrated approach Ronacher et al. (61) identified a six-marker model consisting of time to positivity (TTP), body mass index (BMI), TNF-β, sIL-6R, IL-12p40 and IP-10 measured at baseline, which predicted relapse with a 75% (95% CI: 0.38–1.0) sensitivity and a 85% (95% CI: 0.75–0.93) specificity in the discovery cohort and 83% (95% CI 0.58–1) sensitivity and 61% (95% CI 0.39–0.83) specificity in the validation cohort (61). Cilliers et al. (62) investigated the early TB treatment response of patients who subsequently remained cured or who relapsed. They investigated the use of individual and multi-marker models to predict treatment outcome and responder classification. Top performing multi-variable models at diagnosis using unstimulated values predicted outcome at 24 months after treatment completion with a sensitivity of 75.0% (95% CI, 42.8–94.5%) and specificity of 72.4% (95% CI, 52.8–87.3%) in leave-one-out cross validation. Month two treatment responder classification was correctly predicted with a sensitivity of 79.2% (95% CI, 57.8–92.9%) and specificity of 92.3% (95% CI, 64.0–99.8%) (62).

## Mtb Antigens With Potential to Differentiate Between the Stages of TB Infection

A determinant for immunodiagnostic test sensitivity, specificity and predictive potential is the antigen(s) that is used for stimulation (42). Mtb responds to the conditions encountered in the host and is therefore hypothesized to express different antigens during different phases of infection. These include DosR regulon encoded antigens, TB reactivation antigens, resuscitation promoting factors (rpfs) and starvation-induced proteins. To date, however, none of these antigens, single or in combination have the ability to discriminate active TB or LTBI with 100% accuracy (58). A study done by Chegou et al. (50) assessed the diagnostic potential of 118 Mtb antigens in TB patients and HHCs in a high-TB burden population. Using a 7-day diluted whole blood assay (WBA) they screened the antigens to assess their diagnostic potential for identifying active TB. The antigens that could distinguish between active TB and LTBI were the rpfs (Rv0867c, Rv2389c, Rv2450c, Rv1009 and Rv1884c), with AUCs between 0.72 and 0.80. A combination of Mtb specific ESAT-6/CFP-10 fusion protein, Rv2624c and Rv0867c accurately predicted 73% of the TB patients and 80% of the non-TB cases after cross validation (50). Loxton et al. (57) studied the ability of Mtb surface protein, heparin-binding hemagglutinin (HBHA) to induce multiple cytokines in peripheral blood mononuclear cell (PBMCs) and whole bood from TB index cases and HHCs (57). HBHA is a 28 kD heparin-binding protein produced by Mtb and *Mycobacterium bovis* (63), present at the outermost layer of the cell and mediates the bacteria-epithelial cell interaction (63, 64). Induction of multifunctional INF-γ-, IL-2-, and IL-17-coexpressing CD4+ T cells was observed in HHCs, but not in active TB cases (57).

Using a sera from 561 TB suspects, Kunnath-Velayudhan et al. (65) found that only about 10% of the Mtb proteome is recognized by antibodies in these individuals; a protein subset that is enriched for secreted and membrane proteins (65). Although an association was observed between the antibody response and the bacterial burden in patients with active disease, the relative response to different antigens varied between individuals. It is currently unclear if the level of antigen expression correlates with relative antigen responses. A study investigating the antibody response to LAM and 7 mycobacterial protein antigens (ESAT-6, Tpx, PstS1, AlaDH, MPT64, 16kDa and 19kDa) and 2 antigen cocktails (TUB: PstS1, 16kDa and APA; TB-LTBI: Tpx, L16) found that individually anti-16 kDa IgA and anti-MPT64 IgA were best able to differentiate between LTBI active TB disease (66). In combination anti-TB-LTBI IgG, anti-Tpx IgG, anti-MPT64 IgA and anti-LAM IgA classified both groups (TB disease or LTBI) with an accuracy of 100% in the re-substitution classification matrix and an accuracy of 95.2% after leave-one-out cross validation. Using antigen combinations may therefore be useful to overcome the variation observed between individuals.

## The Diagnostic Potential of Host Iron Metabolism Indicators

Perturbations in host iron homeostasis due to inflammation occurs following infection with a range of pathogens and indicators of iron metabolism may therefore lack the specificity required for diagnostic biomarkers. A recent study investigating the etiology of community-acquired pneumonia demonstrated that ferritin levels could distinguish between atypical and typical bacterial agents (odds ratio [OR], 2.26; 95% CI, 1.18–4.32; p = 0.014), while hepcidin and ferritin distinguished between atypical bacterial and viral etiology (hepcidin: OR = 3.12, 95% CI = 1.34–7.28, p = 0.008; ferritin: OR = 2.38, 95% CI = 1.28–4.45, p = 0.006) (67). The authors proposed that these differences may be due to the intracellular location of atypical bacteria, and unique aspects of the response to viruses, suggesting that host-pathogen interactions are reflected to some extent by iron metabolism indicators.

Recent studies have investigated using iron metabolism indicators in combination to improve specificity. Dai et al. (68) built a TB prediction model using three iron biomarkers (serum iron, ferritin and transferrin levels) and a training set which included PTB, HC, HHC, LTBI and cured patients. Validation on an independent cohort showed that the model successfully differentiated TB from non-TB, with 77% sensitivity (95% CI 74 to 80), 92% specificity (95% CI87 to 96), 98% PPV (95% CI 97 to 99) and 40% NPV (95% CI 35 to 45) (68). Another approach to improve specificity is to use iron metabolism indicators in combination with Mtb-specific biomarkers. A predictive model to discriminate between TB and LTBI was developed by Luo et al. (69), using iron metabolism indicators (serum ferritin, serum iron, transferrin, total iron binding capacity, unsaturated iron binding capacity, and soluble transferrin receptor) in combination with the T-spot assay (TBAg/PHA ratio). Validation of the model on an independent cohort showed an AUC of 0.965 (95% CI, 0.934–0.997) with 92.42% sensitivity and 90.57% specificity (69).

Given that host iron status impacts TB disease severity, indicators of host iron metabolism may be useful for predicting disease progression. Panda et al. observed plasma iron and hemoglobin levels were low in PTB patients compared to HHC and HCs, suggesting anemia of inflammation due to active disease (21). In an earlier study, Minchella et al. (70) observed higher ferritin and hepcidin concentrations among early TB-progressors compared to delayed progressors (mean ferritin 50.2 *vs.* 26.2 ng/ml; p = 0.027; mean hepcidin 37.7 *vs.* 5.6 ng/ml; p = 0.036), while low transferrin around the time of Mtb exposure was identified as a risk factor for all progressors (70). The authors speculated that higher ferritin and hepcidin levels provide intracellular bacteria with increased access to iron, resulting in increased replication and active disease.

## The Diagnostic Potential of Mtb Antigens Induced by Iron Limitation

Given that Mtb must compete with the host for iron during infection, it is conceivable that Mtb proteins involved in maintaining iron homeostasis may have utility as biomarkers. Sivakolundu et al. (71) showed high levels of anti-HupB antibodies in TB patients compared with HHC and HCs (71). In a follow up study, Sritharan et al. (28) observed a significantly higher level of anti-rHupB-F2 (aa 63–161) antibodies in all groups with TB compared to controls (p < 0.05), including relapse and EPTB patients (28). Both studies showed a negative correlation between serum iron levels and the titre of anti-HupB antibodies in the active TB groups, suggesting that HupB may be induced in response to iron limitation in these patients (28, 71).

In addition to iron-acquisition gene transcripts, iron-starved Mtb upregulates iron-sulphur (Fe-S) cluster biosynthesis genes (*sufR-sufB-sufD-sufC-csd-nifU-sufT*) (**Figure 1B**) and several Fe-S containing proteins. Fe-S biogenesis therefore seems to be prioritized by Mtb in its iron sparing strategy, to ensure that essential Fe-S cluster proteins remain functional (72). Induction of the *sufR-sufB-sufD-sufC-csd-nifU-sufT* operon also occurs in response to oxidative stress and intracellular growth (73, 74), and transcription is regulated by the Fe-S containing protein SufR (75, 76) Kumar et al. (77) detected anti-SufR antibodies in serum of TB patients suggesting that this regulator is expressed in Mtb during growth within the human host (77). This was further supported by the observation that *sufR* expression was induced in Mtb in sputum from TB patients, relative to expression *in vitro* (77). Rv2204c is a A-type carrier protein predicted function in Fe-S cluster biogenesis (78). A study investigating antigen-specific memory and regulatory T cells found a higher proportion of Rv2204c reactive CD4^+^ Treg in active TB patients than LTBI individuals (79). The finding is however preliminary given the small number of individuals included in the study (15 per group).

The observation by Pisu et al. (31) that Mtb may be experiencing different levels of iron limitation in different cell types raises an important question for biomarker discovery; if bacteria reside in different niches within the host, how would this influence the global immune response, and by implication the choice of diagnostic antigens? Yang et al. (80) assessed BfrB-specific cellular responses in human cohorts, including HCs, LTBI and PTB patients and showed that BfrB is capable of inducing moderate cellular responses in TB patients and LTBI individuals (80). Interestingly, BfrB-specific IFN-γ release in PTB patients was lower than for ESAT-6 or CFP-10 stimulation, while it was comparable in the LTBI cohort, suggesting that BfrB becomes immunodominant during latent infection. Similar trends have been observed for dormancy-associated antigens (81, 82), suggesting that the comparing immunodominance of different antigens may allow discrimination between disease stages.

## Conclusion

The shortcomings of current immunodiagnostics for the detection of progression from LTBI to active TB, diagnosis and treatment monitoring of TB highlights the need for new TB biomarkers. Performance targets for point-of-care tests in adults with smear-positive pulmonary TB include a sensitivity and specificity of 95%, while sensitivity in smear-negative cases should attain 60-80% and specificity of 95%. It is difficult to predict which biomarkers will attain this level of discrimination, however given the importance of host and bacterial iron metabolism during infection we believe this area warrants further investigation for TB biomarker development.

## Author Contributions

MW and AL conceptualized the study. LB wrote the manuscript with support from AL and MW. AL and MW edited the manuscript and gave critical comments. All authors contributed to the article and approved the submitted version.

## Funding

This work was supported by the South African Medical Research Association (SAMRC), Centre of Excellence for Biomedical Tuberculosis Research (CBTBR). MW was supported by a research career award from the National Research Foundation (Grant number: 91424).

## Conflict of Interest

The authors declare that the research was conducted in the absence of any commercial or financial relationships that could be construed as a potential conflict of interest.

## Publisher’s Note

All claims expressed in this article are solely those of the authors and do not necessarily represent those of their affiliated organizations, or those of the publisher, the editors and the reviewers. Any product that may be evaluated in this article, or claim that may be made by its manufacturer, is not guaranteed or endorsed by the publisher.
